# A second hereditary cancer predisposition syndrome in a patient with lynch syndrome and three primary cancers

**DOI:** 10.1186/s13053-024-00281-9

**Published:** 2024-06-12

**Authors:** Annmarie Taheny, Haylie McSwaney, Julia Meade

**Affiliations:** 1 Department of Medical Genetics & Genomics, UPMC Children’s Hospital of Pittsburgh, Pittsburgh, PA USA; 2 Department of Pediatrics, University of Pittsburgh School of Medicine, Pittsburgh, PA USA

**Keywords:** Hereditary cancer conditions, Case report, Lynch syndrome, CHEK2 hereditary cancer predisposition, Multiple hereditary cancer conditions, NCCN guidelines

## Abstract

Current National Comprehensive Cancer Network ® (NCCN ®) guidelines for Colorectal Genetic/Familial High-Risk Assessment provide limited guidance for genetic testing for individuals with already diagnosed hereditary cancer conditions. We are presenting the case of a 36-year-old woman who was diagnosed with Lynch Syndrome at age 23 after genetic testing for a familial variant (c.283del) in the *MLH1* gene. The patient had a previous history of Hodgkin Lymphoma at the time of familial variant testing, and she would later develop stage IIIa cecal adenocarcinoma at age 33 and metastatic papillary thyroid carcinoma at age 35. The patient’s family history included a first-degree relative who was diagnosed with colorectal cancer at age 39, multiple second-degree relatives with colorectal, endometrial, and stomach cancer, and third and fourth-degree relatives with breast cancer. In light of her personal and family history, a comprehensive cancer panel was recommended. This panel found a second hereditary cancer predisposition syndrome: a likely pathogenic variant (c. 349 A > G) in the *CHEK2* gene. This specific *CHEK2* variant was recently reported to confer a moderately increased risk for breast cancer. The discovery of this second cancer predisposition syndrome had important implications for the patient’s screening and risk management. While uncommon, the possibility of an individual having multiple cancer predisposition syndromes is important to consider when evaluating patients and families for hereditary cancer, even when a familial variant has been identified.

## Introduction

An estimated 5 to 10% of all cancers are associated with genetic variants in cancer predisposition genes [1]. Current NCCN guidelines for Colorectal Genetic/Familial High-Risk Assessment (2.2023) recommend testing family members for familial variants and considering multigene panel testing for individuals with negative genetic testing results whose personal or family histories are strongly suggestive of an inherited susceptibility [2]. However, the NCCN guidelines provide limited guidance on genetic testing for individuals who have already had positive genetic testing results or individuals whose history is not adequately explained by the familial variant [2].

## Case presentation

The 36-year-old patient presented to the Cancer Predisposition Clinic due to a recent diagnosis of metastatic papillary thyroid carcinoma at age 35. The patient had a previous history of Hodgkin Lymphoma diagnosed at age 17, stage IIIa cecel adenocarcinoma diagnosed at age 33, and a pathogenic variant in the *MLH1* gene (c.283del). The patient was interested in further genetic testing to assess her future cancer risk if she underwent radiation exposure for her thyroid cancer. Her family history was taken at this appointment and the Blueprint Genetics Comprehensive Hereditary Cancer Panel was recommended.

**Fig. 1:**
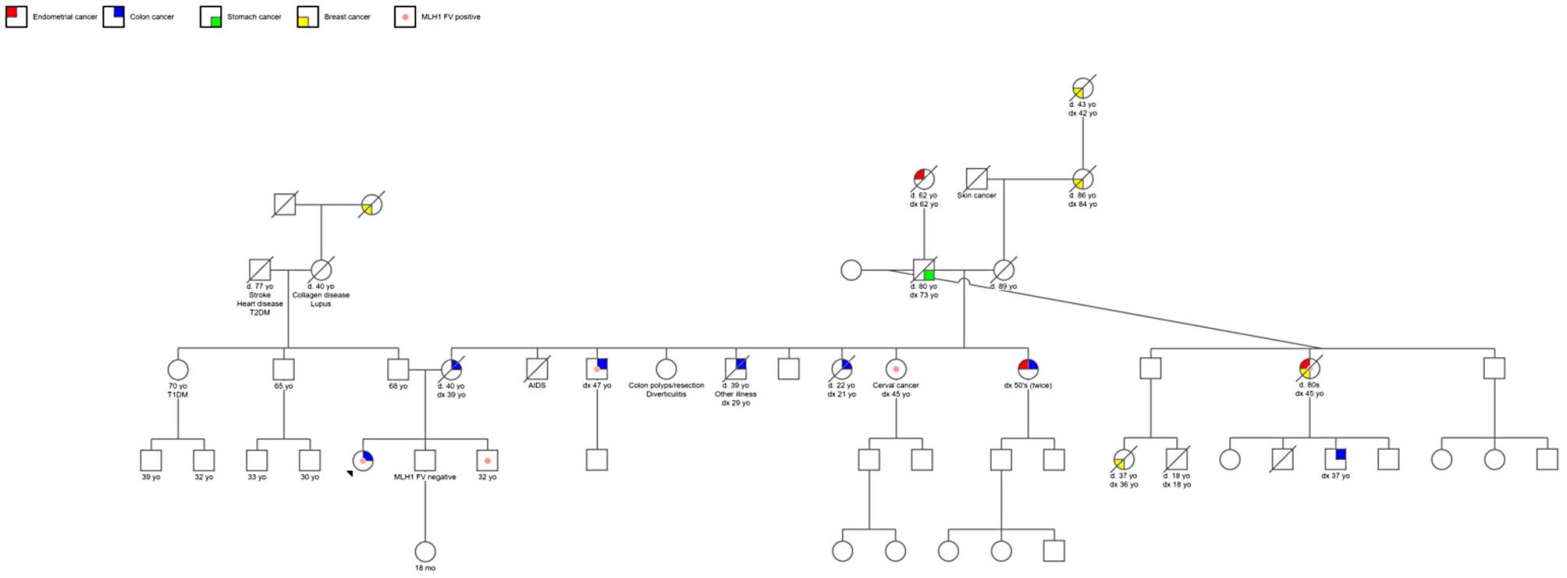
Family pedigree demonstrating multiple first and second-degree relatives with both Lynch Syndrome (LS) and non-LS associated cancers

The patient’s family history was significant for multiple family members with cancer (Fig. 1). The patient’s maternal family history was positive for multiple first and second degree relatives affected by cancers that are traditionally associated with Lynch syndrome, as well as multiple third degree relatives affected by breast cancer, which is not currently associated with Lynch syndrome due to insufficient evidence [1]. The pedigree showed one cousin with a similar age of onset of cancer with our patient, but most family members developed cancer in their late thirties and beyond. Our patient had an unusual presentation compared to her family members due to her age of onset and the types of cancer she developed. Her paternal family history was positive for breast cancer, but otherwise was unremarkable.

## Results

The Comprehensive Hereditary Cancer panel returned positive for two likely pathogenic variants: the previously identified familial *MLH1* variant (c.283del), and a newly identified *CHEK2* variant (c.349 A > C). No other pathogenic, likely pathogenic, or variants of uncertain significance were found in the 160 genes evaluated. Parental testing was not completed, and while we know the *MLH1* variant is maternally inherited, we do not know the inheritance of the *CHEK2* variant at this time. *CHEK2* pathogenic variants convey a moderately increased risk for both breast cancer and colorectal cancer, and with both her maternal and paternal family history of breast cancer it is difficult to assess which side of the family this variant may have come from without further parental testing [2].

## Discussion

While the *CHEK2* pathogenic variant does not fully explain her Hodgkin Lymphoma, it may partially explain her cecal adenocarcinoma diagnosis a decade before the estimated average presentation for colorectal cancer (at 44 years old) in *MLH1*-associated Lynch syndrome [2]. This *CHEK2* pathogenic variant also allows us to better assess her risk for breast cancer, as the ACMG published a practice resource that found her specific *CHEK2* pathogenic variant provides a moderate risk for breast cancer, which would not have been addressed if her management was only guided by the *MLH1* pathogenic variant recommendations [2, 3].

This patient’s case is important due to her unusual presentation in the setting of Lynch syndrome and the second hereditary cancer condition that was found on her genetic testing years after the first hereditary cancer condition was found. Current guidelines focus on familial testing for a known pathogenic variant, with the limited guidance that personal and family history should still be taken into account. Furthermore, the current guidelines do not provide extensive recommendations for further testing in individuals with previous positive genetic testing results. Additionally, as our understanding of hereditary cancer conditions advances, previous positive and negative genetic test results that are a decade or older should be revisited through the lens of our current understanding. We present this case to encourage more thorough genetic testing in already positive patients who have unusual presentations and whose history is not fully explained by previous positive familial variant testing results.

## Data Availability

No datasets were generated or analysed during the current study.
